# Challenges of the Oxycodone Hydrochloride Shortage

**DOI:** 10.3390/reports9020189

**Published:** 2026-06-17

**Authors:** Gursan Gunes Yenidogan, Nagihan Duran Yakar, Enise Alioglu, Salim Taner Gözükızıl, Aysegul Bilen

**Affiliations:** Department of Pain Medicine, Prof. Dr. Cemil Tascioglu City Hospital, 34384 Istanbul, Turkey; drnagihanyakar@gmail.com (N.D.Y.); eniseogras@gmail.com (E.A.); aysegulbilen@gmail.com (A.B.)

**Keywords:** drug shortage, pain management, oxycodone hydrochloride, cancer pain

## Abstract

**Objectives:** To evaluate the clinical impact and treatment adaptations during the immediate-release oxycodone hydrochloride shortage. **Methods:** This retrospective, observational study was conducted during the oxycodone shortage period (May 2024–March 2025) in patients with cancer pain. Pain intensity was assessed using the Numerical Rating Scale (NRS) at baseline (prior to switching, while receiving oxycodone) and at follow-up (after switching to alternative analgesics). Changes in pain intensity were evaluated using within-patient differences (ΔNRS), with clinically meaningful worsening defined as an increase of ≥2 points. Descriptive and inferential statistics were used to summarize patient characteristics and outcomes. **Results:** Of 300 patients screened, 55 met inclusion criteria (mean age 65.2 ± 11.0 years; 63.6% male). Pain intensity increased significantly following treatment modification during the period of oxycodone unavailability, with mean NRS scores rising from 4.3 ± 1.7 to 5.9 ± 2.5 (*p* < 0.001). The mean ΔNRS was +1.56 (95% CI 0.79–2.34), with clinically meaningful worsening observed in 36 patients (65.5%). No statistically significant association was observed between substitute analgesic type and clinically meaningful worsening (*p* = 0.11). **Conclusions:** The oxycodone shortage was associated with worsened pain control and increased need for treatment modifications in cancer patients, highlighting the importance of uninterrupted access to essential opioids.

## 1. Introduction

In 2025, an estimated 2,041,910 new cancer cases are expected to be diagnosed in the United States, according to projections by the American Cancer Society and the National Center for Health Statistics [1]. The prevalence of pain has been reported as approximately 33% among cancer survivors after curative treatment and as high as 64% among patients with metastatic or terminal disease [2]. Cancer pain remains one of the most common and burdensome symptoms associated with malignancy and may substantially impair quality of life, functional status, and overall well-being. Cancer-related pain arises through multiple mechanisms, including direct tumor invasion, treatment-related tissue injury, inflammation, and neuropathic processes, often resulting in a complex pain phenotype [3,4,5]. International guidelines, including those issued by the World Health Organization (WHO), emphasize that the primary goal of cancer pain management is to reduce pain to a level that allows an acceptable quality of life. Guideline-based management recommends selecting analgesic therapy according to pain severity, with opioid analgesics constituting a cornerstone of treatment for patients with moderate-to-severe cancer pain. Furthermore, the WHO highlights that analgesics, including opioids, should be both available and accessible to patients who require them, underscoring the importance of maintaining uninterrupted access to essential pain medications [6].

Among the available opioid analgesics, oxycodone is one of the most widely used agents in cancer pain management. The immediate-release formulation of oxycodone has been approved by the U.S. Food and Drug Administration (FDA) for the treatment of acute or chronic moderate-to-severe pain in patients for whom opioid therapy is deemed appropriate and when alternative pain management strategies are insufficient [7].

Immediate-release oxycodone is widely used for the management of breakthrough cancer pain and other situations requiring rapid analgesic adjustment, making its availability particularly important in routine cancer pain practice [8]. In Turkey, access to rapid-onset opioid formulations is more limited than in many other countries, as transmucosal fentanyl products (e.g., sublingual, buccal, and intranasal formulations) are not available. Consequently, immediate-release oxycodone and morphine remain the principal short-acting opioid options in routine clinical practice. Therefore, the unavailability of immediate-release oxycodone represents the loss of one of the few available therapeutic alternatives for the management of cancer pain.

Due to disruptions in supply chains, global crises in raw material procurement, and limitations in manufacturing capacity, periodic shortages of oxycodone hydrochloride (Oxycodone HCl) have been reported in many countries [9,10,11]. In settings where rapid-onset strong opioid options are limited, the unavailability of immediate-release oxycodone may substantially restrict treatment flexibility and compel clinicians to perform opioid rotation using a narrow range of available alternatives. However, there are only a limited number of studies in the literature that have addressed the clinical approaches employed during periods of oxycodone shortage and their impact on patient outcomes. In this context, our study aimed to highlight the clinical importance of immediate-release oxycodone in cancer pain management and to describe the challenges and treatment modifications encountered during its unavailability.

## 2. Materials and Methods

This retrospective, observational study was conducted at the Department of Pain Medicine, Prof. Dr. Cemil Taşcıoğlu City Hospital, Istanbul, Turkey. The study was approved by the Clinical Research Ethics Committee of Prof. Dr. Cemil Taşcıoğlu City Hospital (approval number: 138; date: 14 April 2025).

Although no official nationwide notification regarding the shortage of Oxycodone HCl was issued in Turkey, a marked shortage was observed between May 2024 and March 2025. Drug unavailability (shortage) was operationally defined at the patient level as the inability to obtain prescribed oxycodone hydrochloride from pharmacies across different cities, with confirmation of its absence through inter-pharmacy communication networks and supply inquiries to pharmaceutical warehouses. Only patients whose opioid switch was explicitly attributed to oxycodone unavailability, rather than clinical factors such as adverse effects, insufficient analgesia, or disease progression, were included in the analysis.

All patient records were independently reviewed by two researchers, and cross-validation was performed to minimize information bias. Opioid rotation strategies were identified from clinical practice and were based on standard equianalgesic conversion tables in accordance with WHO guidelines for the pharmacological management of cancer pain [6]. In routine practice, the calculated equianalgesic doses were generally reduced to account for incomplete cross-tolerance and subsequently individualized and titrated according to clinical response [12].

Of the 300 patients screened, 240 were excluded because they were already receiving analgesic regimens other than immediate-release oxycodone hydrochloride at baseline and therefore were not affected by a shortage-related interruption of oxycodone therapy. An additional 5 patients were excluded because they did not attend the follow-up visit, resulting in missing outcome data. Thus, 55 patients met the eligibility criteria and were included in the final analysis. Eligible patients were those who had received regular oxycodone HCl therapy for at least one month before treatment modification due to drug unavailability and had follow-up records documenting pain scores.

Pain intensity data were obtained from patients’ self-reported Numerical Rating Scale (NRS, 0–10) scores as documented in the medical records. Baseline values reflected current pain intensity reported by patients at the time of clinical evaluation during ongoing oxycodone therapy, whereas follow-up values corresponded to the outpatient visit approximately two weeks after switching to alternative analgesics due to oxycodone unavailability. At the follow-up visit, treatment adjustments, including the initiation of add-on therapy, were made based on clinical status. Add-on therapy was defined as the addition of a new analgesic agent, typically from a different pharmacological class, when clinically indicated.

**Figure 1:**
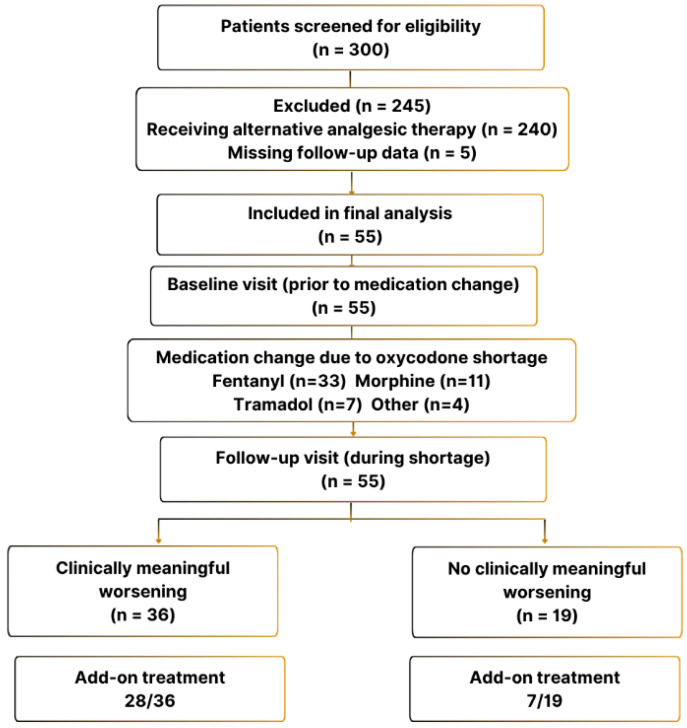
Flow chart of the study design.

Changes in pain intensity were evaluated using within-patient differences (ΔNRS). The primary outcome was clinically meaningful worsening in pain intensity at the follow-up visit, defined as an increase of ≥2 points in the NRS score compared with baseline, consistent with established thresholds for minimal clinically important differences. Patients who did not meet this criterion were considered not to have clinically meaningful worsening. Independent variables included demographic and clinical characteristics such as age, sex, primary diagnosis, metastatic status, and the type of substitute drug (e.g., fentanyl, morphine sulfate, tramadol, other). The secondary outcome was the requirement for add-on treatment. The study flow is presented in Figure 1.

### Statistical Analysis

Statistical analyses were performed using appropriate statistical methods. Categorical variables were presented as frequencies and percentages, and continuous variables as mean ± standard deviation (SD) or median (interquartile range, IQR), as appropriate. Comparisons between categorical variables (e.g., clinically meaningful worsening in pain intensity and type of substitute medication, sex, or metastatic status) were performed using the chi-square test or Fisher’s exact test, as appropriate, given the relatively small and uneven group sizes. Differences between paired continuous variables (e.g., NRS scores at the baseline (pre-shortage) and follow-up visits (during shortage)) were assessed using the Wilcoxon signed-rank test, as the data were not normally distributed (Shapiro–Wilk test). Statistical significance was defined as a two-sided *p*-value < 0.05.

## 3. Results

A total of 300 patients were screened during the study period, of whom 55 met the inclusion criteria and were analyzed. The median age was 64 years (mean 65.2 ± 11; range 39–90). Of these, 35 (63.6%) were male and 20 (36.4%) were female. The most frequent primary tumors were lung (18, 32.7%), gastrointestinal (12, 21.8%), genitourinary (10, 18.2%), and breast (7, 12.7%) cancers, followed by hepatobiliary/pancreatic tumors (2, 3.6%) and a heterogeneous group classified as other malignancies (6, 10.9%). Metastatic disease was present in 40 patients (72.7%), while 15 (27.3%) had no evidence of metastasis (Table 1).

**Figure 2:**
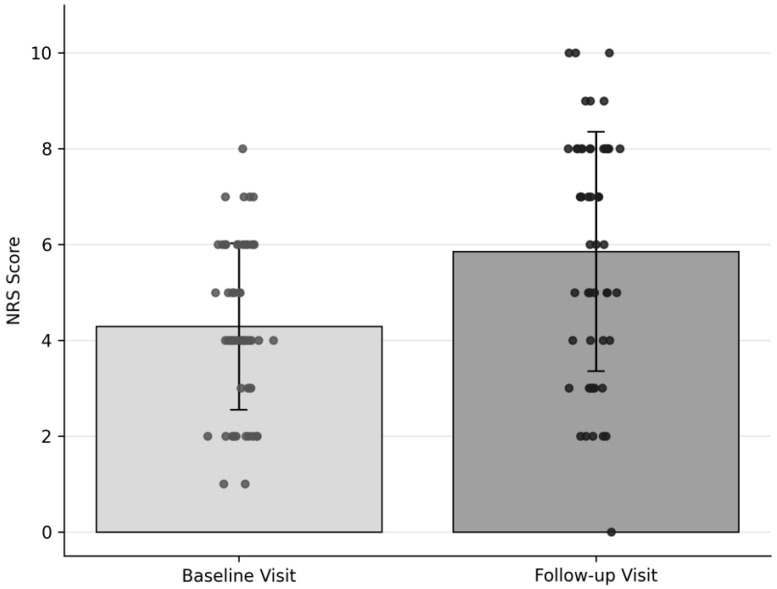
Pain intensity at baseline and follow-up visits. Bars represent mean NRS scores, and error bars indicate standard deviations. Individual points represent NRS scores of individual patients (*n* = 55). Baseline visit scores were recorded during oxycodone treatment, whereas follow-up visit scores were recorded after switching to alternative analgesics due to oxycodone unavailability. Pain intensity was significantly higher at the follow-up visit compared with the baseline visit (Wilcoxon signed-rank test, *p* < 0.001).

The NRS scores during oxycodone treatment were 4.3 ± 1.7 (median: 4), whereas after switching to alternative analgesics due to oxycodone unavailability, the scores at the follow-up visit were 5.9 ± 2.5 (median: 6). This difference was statistically significant (Wilcoxon signed-rank test, *p* < 0.001) (Figure 2).

The mean change in NRS score (ΔNRS) was +1.56 points (95% CI, 0.79–2.34), with a median ΔNRS of 2 (IQR, 0–3). Clinically meaningful worsening (defined as an increase of ≥2 points in NRS) was observed in 36 patients (65.5%).

Clinically meaningful worsening did not differ significantly by sex (*p* > 0.99) or metastatic status (*p* > 0.99). Patients who underwent treatment modification were primarily managed with a fentanyl-based strategy, either through dose escalation in those previously receiving a fentanyl–oxycodone combination or initiation of fentanyl alone (33, 60.0%). Other substitutions included morphine sulfate (11, 20.0%) and tramadol (7, 12.7%), while a small number of patients received non-opioid alternatives. No statistically significant association was observed between substitute medication type and clinically meaningful worsening (*p* = 0.11; Table 2), although subgroup comparisons were limited by small and imbalanced group sizes.

During follow-up, 35 patients (63.6%) required additional medication, while 20 (36.4%) continued without add-on therapy. Add-on treatment was required in 28 of 36 patients (77.8%) with clinically meaningful worsening compared with 7 of 19 patients (36.8%) without worsening. This difference was statistically significant (odds ratio 6.00, 95% CI 1.77–20.31; Fisher’s exact test, *p* = 0.003) (Table 3). Among the 35 patients who required add-on treatment, the adjunctive medications used included NSAIDs (*n* = 13), morphine sulfate (*n* = 8), tramadol (*n* = 8), gabapentinoids (*n* = 4), duloxetine (*n* = 1), and fentanyl (*n* = 1).

## 4. Discussion

### 4.1. Global and National Reports on Oxycodone Shortages

According to recent notifications issued by official health authorities and state-supported professional associations, significant supply problems have been reported for immediate-release (IR) oral tablet formulations of oxycodone hydrochloride, which is widely used in the management of cancer-related pain in the United States during 2024 and 2025. Updated data from the University of Utah Drug Information Service and the American Society of Health-System Pharmacists (ASHP) indicate that the production of several dosage strengths has been discontinued, while others have been made available only in limited and irregular batches. Access difficulties have been particularly pronounced for 15 mg and 30 mg formulations, whereas even lower-dose products have faced prolonged or uncertain resupply timelines [11]. In Australia, the Therapeutic Goods Administration (TGA) reported that certain oral and parenteral formulations of oxycodone hydrochloride were withdrawn from the market between 2023 and 2025 for commercial reasons [5]. Similarly, the Canadian Pharmacists Association (CPhA) and Health Canada announced that, as of 2025, analgesics containing oxycodone or codeine in combination with acetaminophen were experiencing nationwide shortages. These shortages were attributed to production interruptions and increased demand. To mitigate the clinical impact, the CPhA issued guidance documents, including opioid conversion tables and recommendations for alternative analgesic options. The guidance emphasized that opioids should not be abruptly discontinued—especially in chronic users due to the risk of physical dependence—but instead tapered or carefully substituted [9]. In contrast, no official shortage notification has been issued in Turkey regarding oxycodone hydrochloride preparations. Nevertheless, between May 2024 and March 2025, field-level observations indicated a clear shortage: many patients reported difficulties obtaining the drug from community pharmacies, outpatient pain clinics frequently encountered patients unable to access the medication, and wholesalers reported stock inadequacies. Consequently, shortage-related treatment modifications were identified through routine clinical practice, including patient reports corroborated by pharmacy communications and pharmaceutical warehouse inquiries. Although this approach reflects real-world conditions, some degree of ascertainment bias cannot be completely excluded. In addition, the absence of an official shortage notification system may have influenced the identification of affected patients, as supply disruptions could not be anticipated or managed proactively.

The primary aim of the present study was to investigate how these access difficulties translated into clinical practice. During the course of the study, it became evident that shortages were officially reported in the United States, Canada, and Australia, but no similar mechanism was available in Turkey. This observation highlights that medication shortages are not merely local events but global phenomena, underscoring both the importance and the applicability of transparent reporting systems.

### 4.2. Predictability of Drug Shortages

Anticipating which pharmaceutical products are at risk of shortage is of critical importance—not only to ensure continuity of clinical care but also to reduce unnecessary hospital admissions, prevent complications that impose additional costs on the healthcare system, and avoid compounding the multifaceted challenges already faced by patients with cancer.

A previous study analyzing the characteristics of drugs prone to shortages introduced the concept of the product life cycle. In this framework, factors such as therapeutic class, target age group, reimbursement status, and patient population size were identified as key determinants in predicting shortage risk. According to the Anatomical Therapeutic Chemical (ATC) classification, drug shortages have most frequently affected medications targeting the nervous system (ATC: N), with analgesics (ATC: N02) being the most impacted subgroup [12].

Oxycodone hydrochloride, the focus of our study, is classified under N02AA05 as an opioid analgesic. Thus, our findings reflect a meaningful example of shortage-related challenges within the analgesic drug category.

In product life cycle analyses, shortages are evaluated with respect to their duration, frequency, and the specific active substances affected across the stages of market introduction, growth, maturity, and decline. It has been demonstrated that drugs that have long been available on the market, are widely prescribed, and are covered by reimbursement schemes are particularly vulnerable to shortages during the maturity and decline stages of their life cycle [12]. In Turkey, oxycodone hydrochloride has been present in the pharmaceutical market for an extended period, is routinely prescribed, and is directed toward a relatively stable patient population. Moreover, it is supported by reimbursement under the National Social Security Institution’s Health Implementation Guideline. These characteristics suggest that the product is currently in the maturity stage of its life cycle. However, its availability in only a single commercial formulation and lack of market competition may contribute to reduced innovation or production investment. This combination may signal a transition toward the decline stage, thereby increasing its susceptibility to shortages [13].

Nevertheless, beyond the structural dynamics of the pharmaceutical market, the most important consideration remains the clinical implications of such shortages at the patient level. Accordingly, it is essential to discuss the real-world reflections of these supply disruptions in clinical practice.

### 4.3. Clinical Implications and Management Strategies

Cancer pain management is inherently multimodal and may involve pharmacological treatment, radiotherapy, interventional procedures, palliative care, and psychosocial support depending on individual patient needs [13]. Opioid analgesics remain a cornerstone of treatment for moderate-to-severe cancer pain, and opioid rotation is an established strategy typically used to optimize analgesia or improve tolerability [14]. However, unlike routine clinical practice where opioid switching is guided by patient-specific considerations, the treatment modifications observed in our cohort were primarily driven by an unexpected medication shortage. Such disruptions may affect not only pharmacological pain control but also treatment continuity, patient confidence, and the overall stability of the pain management process [15].

The Global Opioid Policy Initiative (GOPI), which evaluated challenges related to opioid access in cancer pain management, collected survey data from oncology and palliative care clinicians working across Africa, Asia, the Middle East, Latin America, and the Caribbean. The survey was designed to assess whether patients with a valid prescription were able to obtain opioid medications in practice, even when these medications were formally approved and available within the country, as well as the accessibility of centers and pharmacies capable of dispensing opioids for cancer pain management. The GOPI study reported that only 7.5% of the world’s population resides in countries with adequate opioid consumption levels, highlighting substantial global inequalities in access to strong opioids used for cancer pain treatment [16].

Notably, oxycodone was included in the 2025 WHO Model List of Essential Medicines for the first time. Although the reasons underlying this decision are beyond the scope of the present study, its inclusion highlights the recognized importance of oxycodone in pain management and underscores the need to ensure reliable access to essential opioid analgesics for patients with cancer pain [17].

Supply disruptions have had notable consequences at the clinical level, particularly by necessitating practices such as opioid rotation. At the time of writing, the pharmaceutical opioid formulations available in Turkey include codeine-containing combination products, tramadol tablets, oxycodone hydrochloride tablets, morphine sulfate tablets, injectable morphine sulfate solution, transdermal fentanyl, and injectable fentanyl solution. Importantly, several formulations that are widely used in many other countries are not available in Turkey. These include controlled-release oral oxycodone, hydromorphone, oxymorphone, methadone, oral transmucosal fentanyl, and hydrocodone. The absence of these formulations considerably limits the options for opioid rotation. During the period of oral oxycodone shortage in Turkey, clinicians were left with only two practical alternatives among strong opioids: transdermal fentanyl and oral morphine sulfate. This restriction not only reduced flexibility in tailoring pain management strategies but also posed challenges in maintaining stable analgesia during treatment transitions.

In our cohort, 24 of 55 patients (43.6%) were receiving a multimodal opioid strategy in which transdermal fentanyl and immediate-release oxycodone hydrochloride were administered concurrently. Since oral transmucosal fentanyl is not available in Turkey, the rationale behind this approach was to use long-acting fentanyl for the control of persistent baseline pain, while employing short-acting oxycodone for the management of breakthrough pain episodes. However, the unavailability of immediate-release oxycodone, combined with the absence of transmucosal fentanyl, may have created a therapeutic gap in the management of breakthrough pain [8].

During the oxycodone shortage, the most frequent management strategy was fentanyl-based treatment (*n* = 33, 60.0%) (Table 2). Among these patients, 24 (72.7%) required escalation of their previously prescribed fentanyl dose. The predominance of elderly individuals in our cohort may have influenced this clinical decision. Fentanyl undergoes minimal hepatic and renal elimination, offering a potentially safer pharmacological profile compared with agents prone to metabolite accumulation. In this context, increasing the fentanyl dose may represent a pragmatic approach to maintaining treatment safety.

In our study, during the period of oxycodone unavailability, morphine sulfate was used as an alternative treatment in 11 patients (Table 2). A Cochrane systematic review evaluating controlled-release (CR) oxycodone versus CR morphine in adults with cancer pain reported that both opioids provided comparable analgesic efficacy, with no statistically significant differences in their adverse effect profiles [18]. In line with these findings, the transition to morphine sulfate observed in our cohort can be considered a pharmacologically appropriate and evidence-based approach for pain management.

As shown in Table 2, no statistically significant association was observed between substitute medication type and clinically meaningful worsening of pain intensity (*p* = 0.11). However, subgroup comparisons should be interpreted with caution due to small and imbalanced group sizes. Furthermore, no a priori power calculation was performed for this secondary analysis, and the study may have been underpowered to detect clinically meaningful differences between treatment groups. Therefore, the absence of a statistically significant association should not be interpreted as evidence of equivalence between substitute analgesic strategies. Across all treatment groups, clinically meaningful worsening was frequently observed. This finding may reflect both the limited sample size and the challenges in maintaining stable analgesia during the period of oxycodone unavailability. The clinical difficulty may have been related to the abrupt discontinuation of oxycodone and the need for unplanned treatment modifications, rather than differences in the intrinsic efficacy of individual agents.

At follow-up after opioid rotation, clinically meaningful worsening was observed in 36 patients (65.5%), while 19 patients (34.5%) did not exhibit such worsening (Table 3). Among patients with clinically meaningful worsening, 28 (77.8%) required additional medication, whereas 8 (22.2%) were managed without add-on therapy. In contrast, among patients without worsening, 7 (36.8%) required additional medication, while 12 (63.2%) did not. This difference was statistically significant (*p* = 0.003).

The observed worsening in pain intensity may also be related to interindividual variability in opioid responsiveness. Differences in opioid pharmacodynamics and receptor-level responses may contribute to reduced analgesic efficacy following opioid conversion, despite the application of equianalgesic dosing principles [19,20,21].

Disease progression, the development of new metastatic lesions, changes in oncologic treatment, and psychological distress may also have contributed to the observed changes in pain intensity. Among these factors, modifications in oncologic treatment may also contribute to pain fluctuations through treatment-related adverse effects or transient symptom exacerbation. In some cases, treatment initiation may be associated with tumor flare phenomena, characterized by a temporary worsening of symptoms, including tumor-related pain [5].

Previous studies have demonstrated that advanced disease burden and functional decline are closely associated with increased pain and poorer quality of life in patients with cancer. Furthermore, psychological factors such as depression and emotional distress are common among patients with advanced cancer and have been shown to correlate with pain severity and pain-related suffering [22].

Moreover, worsening pain control may not solely reflect pharmacological or treatment-related factors but could also be influenced by the psychological distress and uncertainty associated with limited drug availability. Opioid shortages have been reported to exert adverse psychosocial effects beyond their direct pharmacological consequences, negatively affecting patients’ trust in treatment, perceived symptom control, and overall quality of life, which may in turn contribute to heightened pain perception [23].

Therefore, although the worsening pain scores observed in our cohort occurred after treatment modifications necessitated by the oxycodone shortage, the contribution of these unmeasured confounding factors cannot be excluded. Because the shortage occurred unexpectedly in routine clinical practice and the study was conducted retrospectively, these variables were not systematically recorded and could not be incorporated into the analysis.

Nevertheless, the primary objective of this study was to evaluate the short-term clinical consequences of an unexpected interruption of ongoing oxycodone therapy due to the drug shortage. To minimize the potential influence of longer-term changes in disease status, pain outcomes were assessed shortly after treatment modification, aiming to capture the immediate clinical impact of oxycodone unavailability.

The limited number of publications in this field underscores the value of our study in providing an important observational perspective both within Turkey and in the global context. The present study retrospectively examines the clinical implications of an unexpected oxycodone shortage in a real-world cancer pain population.

Collaboration among physicians, healthcare administrators, and pharmacists is essential to anticipate potential shortages and to prepare patients within the framework of multidimensional pain management. Such collaboration may facilitate safer and more controlled therapeutic transitions. In light of these findings, ensuring the consistent availability of essential opioid preparations for patients with cancer and expanding the diversity of available agents may help mitigate potential treatment disruptions in the future.

This study has several limitations. First, its retrospective, observational, and single-center design inherently restricts the generalizability of the findings. In addition, due to the retrospective nature of the study, causality cannot be established, and the observed changes in pain intensity should be interpreted as associations rather than direct effects of oxycodone shortage. It should also be noted that drug shortages typically arise abruptly and unpredictably, making prospective data collection and standardized management approaches difficult to implement in real-world settings.

The relatively small sample size further reduced the statistical power, particularly in subgroup analyses by type of substitute analgesic. Furthermore, although pain intensity was assessed using NRS scores, other potential confounders—such as disease progression, recent oncologic interventions, performance status, and concurrent medications—could not be comprehensively evaluated due to incomplete records. These factors may have influenced pain outcomes independently of opioid substitution.

In addition, the follow-up assessment was conducted approximately two weeks after opioid rotation. Assessing outcomes shortly after treatment modification may have reduced the potential influence of time-dependent confounding factors, such as disease progression, changes in oncologic treatment, performance status, and other clinical variables that could affect pain intensity independently of opioid substitution. However, this relatively short follow-up period may not fully reflect long-term analgesic stabilization. Longer-term follow-up data were not available.

Another limitation is that pain outcomes were evaluated exclusively using NRS pain intensity scores. While pain intensity remains a key indicator of analgesic effectiveness, it does not fully capture the broader impact of cancer pain on patients’ quality of life and functional status [3]. Because these outcomes were not systematically documented in the available retrospective records, they could not be evaluated in the present study. Future prospective investigations should incorporate multidimensional outcome measures to better characterize the overall clinical impact of opioid shortages.

Finally, the limited number of published studies specifically addressing opioid shortages made it difficult to conduct a comprehensive literature review and fully contextualize the findings. Despite these limitations, the study provides valuable real-world evidence regarding the potential clinical implications of opioid shortages. Importantly, this study does not aim to critique any specific healthcare system; rather, it seeks to highlight the challenges encountered in clinical practice during unexpected disruptions in medication availability.

## 5. Conclusions

The findings of this study suggest that the oxycodone hydrochloride shortage may be associated with worsening pain control and frequent need for opioid rotation and may have contributed to a greater need for adjunctive therapy. However, these findings should be interpreted in the context of the study’s small and highly selected cohort, which may limit their generalizability to broader cancer pain populations and other healthcare settings. These findings provide real-world evidence of the challenges faced by patients and clinicians during unexpected drug shortages. Continuous availability of essential opioid formulations, greater diversity of analgesic options, and transparent shortage reporting systems may help support continuity of care in cancer pain management.

## Figures and Tables

**Table 1:** Demographic characteristics of the study population.

Characteristic	Value
Age (years), mean ± SD	65.2 ± 11.0
Sex, *n* (%)	
Male	35 (63.6)
Female	20 (36.4)
Primary tumor type, *n* (%)	
Lung	18 (32.7)
Gastrointestinal	12 (21.8)
Genitourinary	10 (18.2)
Breast	7 (12.7)
Hepatobiliary/Pancreatic	2 (3.6)
Other malignancies	6 (10.9)
Metastatic status, *n* (%)	
Metastatic disease	40 (72.7)
No metastasis	15 (27.3)

**Table 2:** Association between substitute medication type and clinically meaningful worsening in pain intensity following oxycodone discontinuation. Data are presented as number of patients (*n*) and percentage (%). Clinically meaningful worsening was defined as an increase of ≥2 points in the NRS score. There were no statistically significant differences between groups in clinically meaningful worsening of pain intensity (chi-square test, *p* = 0.11).

Medication Group	Clinically Meaningful Worsening, *n* (%)	No Clinically Meaningful Worsening, *n* (%)	Total, *n*
Fentanyl-based management	25 (75.8)	8 (24.2)	33
Morphine sulfate	7 (63.6)	4 (36.4)	11
Tramadol	3 (42.9)	4 (57.1)	7
Other (non-opioid alternatives)	1 (25.0)	3 (75.0)	4
Total	36 (65.5)	19 (34.5)	55

**Table 3:** Association between clinically meaningful worsening in pain intensity and requirement for add-on medication. Percentages represent row percentages.

Pain Outcome	No Additional Medication, *n* (%)	Additional Medication, *n* (%)	Total, *n*
No clinically meaningful worsening	12 (63.2)	7 (36.8)	19
Clinically meaningful worsening	8 (22.2)	28 (77.8)	36
Total	20 (36.4)	35 (63.6)	55

## Data Availability

The datasets generated and/or analyzed during the current study are available from the corresponding author upon reasonable request.
